# Midwives’ experiences with simulation-based team training in newborn resuscitation: impacts on clinical practice

**DOI:** 10.1186/s12912-025-03368-3

**Published:** 2025-07-01

**Authors:** Liza Olsen-Bremmeng, Linda Wike Ljungblad

**Affiliations:** 1 Department of Health and Care Sciences, Faculty of Health Sciences, UiT, The Arctic University of Norway, Havnegata 5, Harstad, 9405 Norway; 2 University Hospital of Northern Norway Harstad, St. Olavsgate 70, Harstad, 9406 Norway; 3 Centre for Women’s, Family and Child Health, University of South-Eastern Norway (USN), Box 235, Kongsberg, N-3603 Norway

**Keywords:** Experience, Midwife, Simulation, Systematic text condensation, Team training, Qualitative method

## Abstract

**Background:**

Midwives play a crucial role in newborn resuscitation as they are the primary caregivers during childbirth. Simulation training provides training experience to increase competence to handle such acute situations. However, team training provides safety for midwives, and good collaboration is vital for the patient safety of newborn babies in need of resuscitation.

**Aim:**

This study aimed to describe midwives’ experiences with simulation-based team training in newborn resuscitation and explore its impact on clinical practice. Specifically, the study seeks to highlight how simulation-based team training can enhance teamwork, strengthen professional competencies, improve team communication, and positively influence patient outcomes in neonatal care.

**Methods:**

The study employed a qualitative method with an explorative design. Semi-structured individual interviews were conducted. The data was collected from October to November 2023 with ten midwives working in maternity wards in Norway. The data was analysed using systematic text condensation.

**Results:**

The analysis revealed three main findings: (1) Psychological safety in a familiar team, (2) Learning and improvement and (3) Clear communication.

**Conclusion:**

Our study highlights the critical importance of fostering psychological safety within familiar teams, emphasising how emotional security contributes to effective learning and professional growth. The findings demonstrate that recognising emotional safety leads to more positive learning experiences, fostering competence and confidence in clinical practice. Furthermore, clear communication is identified as essential for collaboration, ensuring beneficial experience in simulation and team training related to newborn resuscitation. By strengthening team communication and psychological security, simulation training not only enhances theoretical understanding but also directly impacts clinical decision-making, patient outcomes, and team dynamics in acute newborn resuscitation scenarios.

**Clinical trial number:**

Not applicable.

## Introduction

According to guidelines from the World Health Organisation (WHO), birth asphyxia is the cause of one in four neonatal deaths worldwide [1]. However, effective ventilation and newborn resuscitation can save lives and reduce the number of newborns who die from birth asphyxia [2]. International and national guidelines emphasise the importance of open airways and adequate ventilation in the 2021 guidelines [3, 4]. Newborn resuscitation is primarily a ventilation task; everyone involved must master this skill [5]. The team is expected to possess the necessary knowledge and competence in technical and non-technical skills, such as team training and communication [4].

Newborn resuscitation is defined as the stabilisation or resuscitation of newborns with insufficient spontaneous respiration who require breathing assistance or, in the worst case, resuscitation with chest compressions and medications [6]. Approximately 10% of all newborns require assistance and stabilisation to breathe on their own after birth, and it is estimated that less than 1% need advanced cardiopulmonary resuscitation [7].

Despite these guidelines, there remains a knowledge gap regarding how teamwork and psychological safety influence the execution of newborn resuscitation. While technical skills are essential, research indicates that non-technical skills—such as communication and collaboration—play a critical role in ensuring optimal patient care [6]. Nevertheless, there is limited research on how simulation-based team training can strengthen these aspects and improve clinical practice [8–10].

This study aims to address this gap by exploring midwives’ experiences with simulation-based team training in newborn resuscitation. By examining how such training enhances teamwork, strengthens professional skills, improves team communication, and positively impacts patient outcomes in neonatal care, the study contributes to a deeper understanding of how structured training programs can better prepare healthcare professionals in acute newborn resuscitation scenarios [8–10].

## Background

In Norway, one of the midwives’ areas of responsibility is the normal birth, with responsibility for both mother and child [11, § 7]. The midwife is responsible for assessing all newborn babies after birth and must decide whether it is necessary to initiate newborn resuscitation [12]. For this assessment, midwives use the Apgar score, a classification of vital parameters, at 1, 5, and 10 min after birth to assess newborn’s condition [13]. In maternity wards in Norway, a midwife may have to initiate newborn resuscitation alone before the rest of the team arrives after being called to help. The midwife’s role in newborn resuscitation is crucial and is just one of the many responsibilities a midwife has in their function [11, § 8].

Experience shows that it is challenging and stressful for midwives to be in acute situations with newborns, as described in recent research that has investigated and concluded that newborn resuscitation is complex and that a supportive work culture is necessary [12]. It is a lifelong learning process starting in midwifery education and continues throughout the professional career [12]. Knowledge, skills, and experience are essential for midwives to be prepared [12]. Frequent simulation training is suggested to improve midwives’ skills and prepare them for newborn resuscitation [14]. The importance of keeping the training as simple as possible with low-dose, high-frequency training in a supportive culture for a more accessible learning process is emphasised [12].

Furthermore, frequent simulation training is recommended due to its potential to impart essential skills in simulation-based education effectively [15]. Hence, other studies demonstrate the necessity of correct skills and competence [16–18]. Skills in newborn resuscitation are underpinned to be best learned and maintained through low-dose, high-frequency simulation training [12, 19].

Communication is paramount for good collaboration, which aligns with similar studies [16, 18]. A study concluded that team training is also beneficial for patients [20]. Another study concluded that clear guidelines and distribution of responsibilities with present leadership improve team training [21]. Moreover, a study assessing knowledge and skills related to simulation and debriefing highlights that advanced communication competencies are required. In particular, the ability to provide constructive feedback without judgment necessitates deliberate practice and skill refinement [22].

Other research has explored simulation training with postpartum haemorrhage [23]. This is comparable to newborn resuscitation regarding how team training can be conducted with the best possible learning for the team, including the importance of learning and reflection, team training, and skills where mistakes are approached at the system level and not at the level of each team member [23]. Newborn resuscitation involves interdisciplinary team training, multiple challenging tasks, and rapid decision-making. Non-technical skills such as teamwork and communication have increasingly become visible and recognised as essential in newborn resuscitation [12, 24]. Notably, according to a systematic review on pediatric simulation-based education, simulation-based learning (SBL) is recognised as a practical pedagogical approach that enhances the competencies and skills of healthcare professionals [25]. The study highlights that simulation facilitates the acquisition of essential skills, including communication, decision-making, attitudes, knowledge, and technical proficiency, within a controlled and immersive learning environment [25].

A knowledge gap was identified; therefore, a need to learn more about how midwives experience simulation and team training as part of their clinical practice. This study highlights the importance of simulation and team training as essential. Furthermore, the focus was on how simulation should be conducted to ensure a positive experience and the factors that can enhance team training. Therefore, this study aims to describe midwives’ experiences from simulation-based team training in the context of newborn resuscitation.

## Methods

This study employed a qualitative method of semi-structured individual interviews.

### Design

The study has an exploratory design, and interviews have been conducted with midwives at various Norwegian maternity wards.

### Recruitment

A convenient sample of midwives was recruited from maternity wards in Norway from the same region because we wanted to investigate midwives’ experiences specifically in maternity wards, as midwives often work alone initially with newborn resuscitation in maternity wards compared to women’s clinics. Maternity wards in a region with long distances were chosen, where access to emergency functions at a women’s clinic takes time and resources. Recruitment was done by emailing department leaders at maternity wards. Information about the study and written consent were provided via a Teams meeting and sent by email before participation. Midwives who wanted to participate contacted the authors themselves by email.

### Inclusion criteria

The inclusion criteria required participants to be registered midwives who were clinically active and had experience with simulation and team training in newborn resuscitation to gather midwives’ experiences from simulation situations and acute scenarios. The purpose was to collect extensive insights from midwives’ experiences in simulation scenarios and acute performance in newborn resuscitation. Ten midwives expressed their interest in participating in the study by email and were thereby included. The midwives were aged between 33 and 54 years and had work experience ranging from 3 to 22 years. All the midwives worked in maternity wards with and without a neonatal intensive care unit, with annual birth numbers ranging from 206 to 381.

### Data collection

A pilot interview via Teams was used to prepare the interviewers and the interview guide before the interviews. This included testing of equipment, the digital interview format, and the adjustment of the interview guide thereafter. Ten semi-structured individual interviews were conducted via Teams from October to November 2023, lasting from 15 to 40 min. Two researchers participated in all the interviews. An interview guide was followed, asking midwives about their experiences with newborn simulation and their experiences as part of a team, as well as advantages, disadvantages, stress, or stress management when performing newborn resuscitation, illustrated in Table 1. In Norway, Norwegian is the primary language spoken by the majority of the population. Accordingly, the data were gathered in Norwegian to maintain linguistic precision and contextual relevance. We utilized a program called Grammarly for translation.

**Table 1:** Interview guide

1. How long have you worked as a midwife? Age?
2. Is the team familiar? How do you experience simulation with newborn resuscitation? Positive or negative experiences?
3. Have simulations affected your competence and confidence as a midwife in acute situations with newborns? Have simulations increased your stress or stress management?
4. Have simulations helped you develop your skills? What significance do you feel that simulation has in real situations? Positive or negative?
5. Do you feel safe or insecure in a simulation? Pros and cons of a familiar team?
6. Have you experienced any situations during simulations that have been challenging for you and your team? How did you resolve these challenges within the team?
7. Have simulations had any impact on communication and collaboration within the team? 8. Can you comment on the teamwork after regular simulations?
9. Familiar team - what significance does it have for you? What significance do you think it has for the team? What advice would you give to other midwives considering implementing regular simulations in their work?
10. Do you feel that the responsible midwife in the delivery room has a different starting point than the rest of the team?
11. What challenges do you see in providing constructive feedback in a familiar team?
12. In what way can the collaboration in the staff group affect the cooperation and communication within the team?
13. Can you tell me about a real event you want to share? Simulation training or real event.

The questions were divided into two parts, with experiences related to simulation and team training, respectively. Based on our considerations, we structured the interview in this manner because we believe that an awareness of one’s own competence facilitates effective teamwork. We intended for the participants to distinguish between their individual simulation experience and their experience within a team context. The questions were formulated neutrally, presented openly, and clarified to the participants that they could address both positive and negative challenges.

Follow-up questions based on the interview guide were asked when needed. The interviews were recorded using an audio recorder and the digital platform Teams, then stored on a secure and approved server at the university and transcribed verbatim. After listening to two interviews, there was a need for follow-up questions, where written experiences were included as supplemental data in the data material as additional information.

### Analysis

Systematic text condensation was chosen to analyse the data material [26]. The analysis method consists of the following four steps where the authors conducted the first three steps separately to ensure the quality of the analysis in collaboration. (1) Total impression - from chaos to themes. We read through the transcribed material continuously to get an overall impression and marked out preliminary themes such as communication, safety, clarity, distributed roles, spinal reflexes, manual skills, performance anxiety, and vulnerability. (2) Identifying and sorting meaning units - from themes to codes. We systematically analysed the text and identified meaning-bearing units, allocating these into code groups. The research question was kept visible throughout the process to remind us of what we sought. (3) Condensation - from code to meaning. We divided each code group into subgroups and attempted to elucidate the meaning using artificial quotes. (4) Synthesizing - from condensation to descriptions and concepts. We summarised into four code groups with condensates from the subgroups [26]. The meaning units were coded and organized into code-groups with sub-groups that exemplified key aspects of each main group. Meaning units within these sub-groups were summarized and condensed. Finally, an analytical text based on the condensed sub-groups was created. The titles were refined and elaborated, and quotations were used to clarify the findings described in Table 2.

**Table 2:**
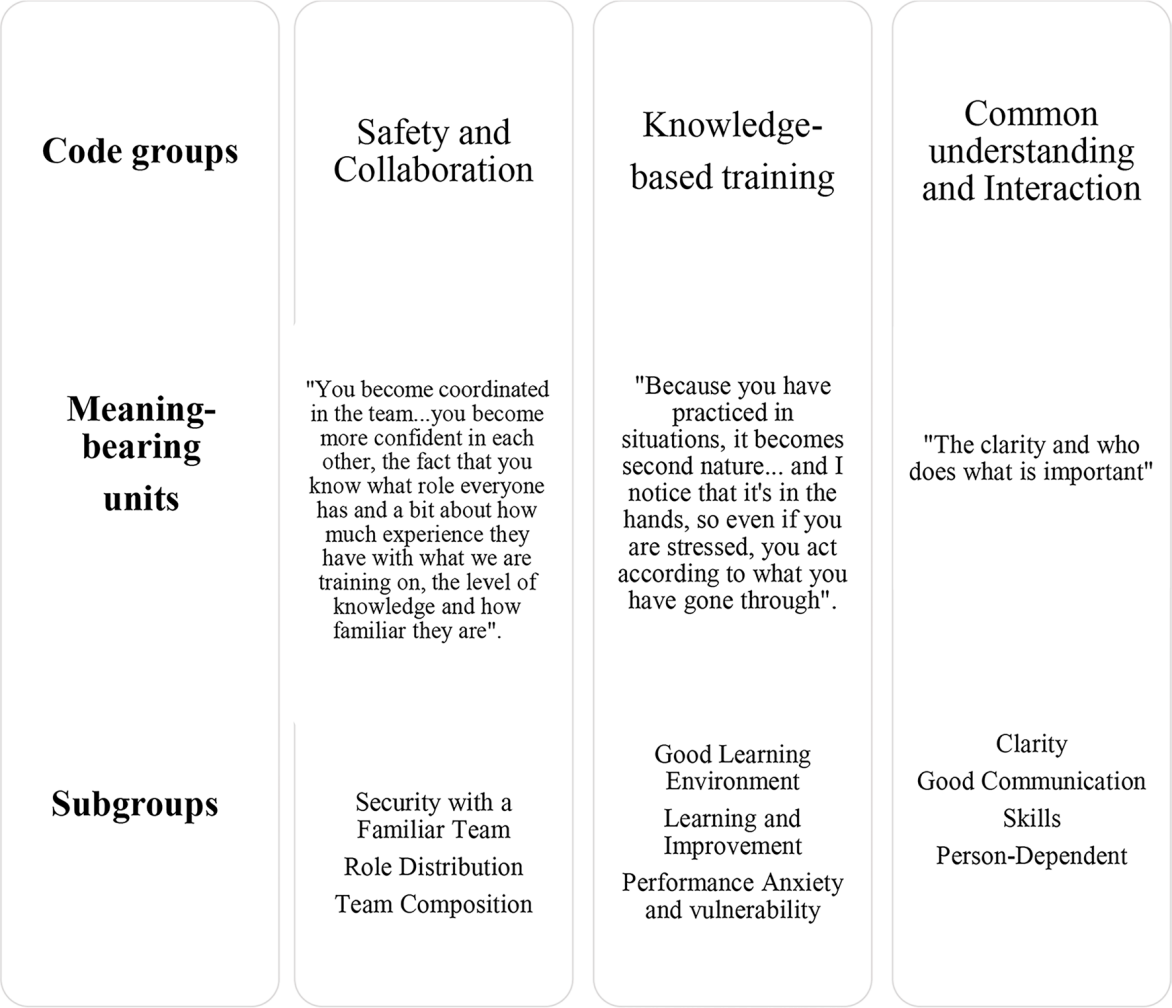
Code groups, Meaning-Bearing units, and subgroups in step four of the analysis

### Trustworthiness

Trustworthiness is assessed through the following criteria: Credibility, Dependability, Confirmability, and Transferability [27]. The credibility was strengthened by including all qualified participants and conducting individual interviews. Dependability was ensured by having midwives of varying ages and experience in maternity care. The authors conducted the analysis separately and together to validate the findings, performed close to the text and collaborated with a supervisor. Transferability was enhanced with detailed descriptions of the context and participants’ characteristics. Consolidated qualitative research (COREQ) reporting criteria were used for transparency and quality [28]. To enhance reflexivity, we engaged in mutual correction among co-researchers throughout the process to remain faithful to the empirical data. Additionally, we utilized individual analysis logs, incorporating self-reflection and ethical considerations consistently throughout the entirety of the process.

### Ethics

The informants received written and oral information about the study, and all read and signed consent forms before the interviews. Participants were given oral information before the interview about their rights, that participation was voluntary, and that they could withdraw until the start of the analysis. The Norwegian Agency for Shared Services in Education and Research (Sikt) assessed that the project complied with privacy regulations (project number 977071). The study was conducted according to the ethical principles of the Helsinki Declaration [22]. Privacy considerations were taken into account during the interviews by not requesting more data than necessary and maintaining respect for each individual’s autonomy and inherent dignity [22].

## Results

The data analysis resulted in three main findings represented by the following categories: (1) Security with a familiar team, (2) Learning and improvement, and (3) Clear communication. The team’s sense of security is strengthened by being well-coordinated, having apparent role distributions, and having well-defined roles where the members are familiar with each other. Learning and improvement as a sub-group are optimised through a good learning arena that creates a spinal reflex in acute situations while reducing the feeling of performance anxiety and vulnerability. Clear and good communication skills are crucial for achieving a common understanding in team collaboration, although this depends on each individual. The result categories are illustrated in Table 3.

**Table 3:**
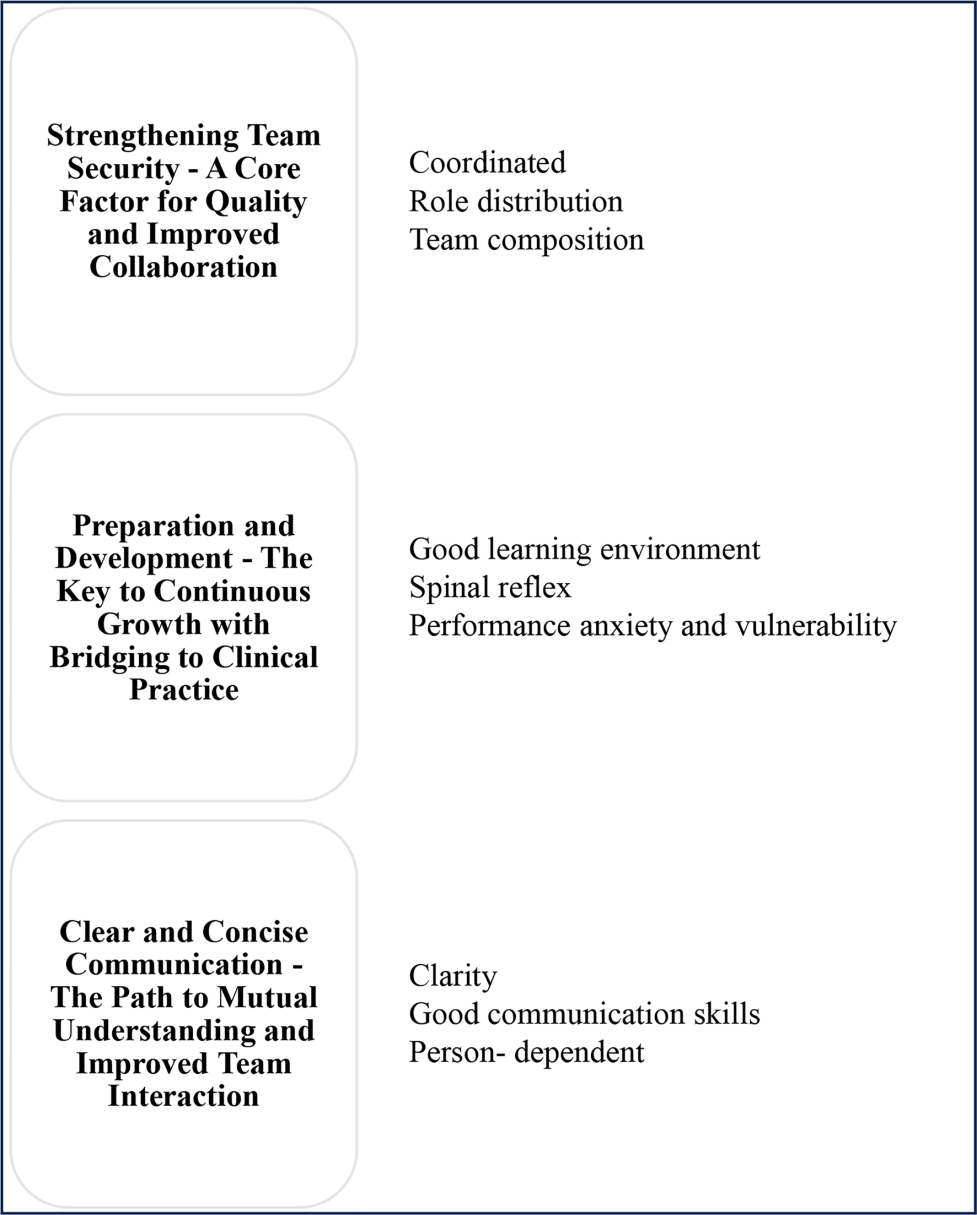
The analysis resulted in three main findings with corresponding subcategories

### Strengthening team security- a core factor for quality and improved collaboration

All the midwives experienced a sense of security with a familiar team. One midwife mentioned that security with a familiar team was critical in acute situations but not transferable in a simulation context. Security was central for most midwives in simulation and team training with newborn resuscitation. The midwives found that they felt safer with a familiar team. One midwife described:

*… but there is something about a familiar team and knowing about each other*,* what one can do*,* and what one has practised together (Informant 1).*

Learning could be less in simulation training in with a familiar team. The sense of security could make the midwives less sharp and unfocused. On the other hand, advocacy was made for a greater degree of low-dose high-frequency simulation with frequent and short simulation training so there would be a short time between training sessions. A midwife described:

*I think that one should have a much greater extent of high-frequency*,* low-intensity simulation*,* that you train often and briefly… this is to make you more confident (Informant 4).*

Midwives reflected on the value of a familiar team and mutual knowledge of each other’s skills and experience; it becomes clear that security within the team plays a decisive role.

#### Coordinated team

The midwives experienced that the team became coordinated, they became more confident in each other, they knew the role everyone had, the level of knowledge of each other, and how much experience the individuals had in newborn resuscitation. A midwife described:

*In a familiar team*,* I feel we work in the same direction (Informant 2).*

A midwife mentioned that the advantages of becoming a coordinated team were that the team knew what to do in acute situations. It was reported that it was safer with familiar colleagues and reassuring to know what the colleagues were capable of. The midwives experienced that unity was significant for safety in simulation training. They spoke about the challenges of giving constructive feedback within a familiar team. However, opinions were divided in this matter. Some believed it was just as difficult to give feedback regardless of the team, while most felt it was easier to give feedback within familiar teams. The midwives mentioned that it was challenging because they did not want it to be perceived as criticism and to maintain a positive collaborative climate among colleagues.

#### Role distribution

The midwives experienced that each team member must be able to fulfil their role for the best possible result, and each individual must be responsible for being prepared for newborn resuscitation. The midwives mentioned that the advantage of a familiar team was that the role distribution was known, and they were aware of each other’s strengths and weaknesses. A midwife described the following:

*It gives me stress management because I know what role I have and what roles the others are supposed to have (Informant 2).*

Based on their experiences, the midwives emphasise that knowing one’s role and the roles of others contributes to stress management, and awareness of role distribution can promote better collaboration in newborn resuscitation.

#### Team composition

Midwives pointed out that the team’s composition was significant for the training, and several midwives experienced this as dependent on each person. A midwife mentioned that insecurity often spreads, especially if the team member who is supposed to know the most feels unsure. The midwives experienced that an unfamiliar team could work. It depended on the personality and experience of each team member, whether the team functioned well or not, and if the team needed to be more familiar. A midwife described it as follows:

*The team has a link that is so weak that the team does not function (Informant 4).*

This description clarifies the importance of the team composition, which can vary for each individual, and underscores the need to strengthen collaboration and improve the team’s efficiency.

### Preparation and development- the key to continuous growth with bridging to clinical practice

The midwives experienced that experience and competence were primarily built on what is learned and improved through simulation training at maternity wards in Norway. The midwives described the following:

*We don’t necessarily face emergencies that often*,* so it is about getting to practice on emergencies frequently*,* and we become good without necessarily being in real situations that often (Informant 8).*

*My experience is mainly based on what I have learned through the simulation (Informant 9).*

The midwives’ experiences emphasise the importance of simulation training, as their experiences are largely based on simulated situations rather than real-life resuscitations.

#### Good learning environment

All the midwives who experienced simulation training found it to be a good learning environment and a positive preparation for acute situations in newborn resuscitation. They recommended simulation training to improve safety and increase newborn resuscitation confidence. A midwife described it as follows:

*The more we train*,* the more confident we become in knowing what to do (Informant 1).*

Within the safe framework of national guidelines for stabilising and resuscitating newborns, the midwives expressed predictability and a good learning environment. All the midwives discussed regular monthly and annual training sessions; however, none felt they trained too often.

Midwives at maternity wards with neonatal intensive care units and paediatricians reported that they still trained regularly even with all available expertise. They believed it was important that everyone in the team was qualified, regardless of the individual’s expertise, and that simulations should be tailored to the actual competence level available on different shifts. This is to make the reality that midwives experience in maternity wards as realistic as possible. A midwife described it as follows:

*When we train*,* everyone is hands-on … there are too many people and hands everywhere in planned simulation. The opposite is in a clinical situation*,* then it feels like you are completely alone (Informant 9).*

The midwives discussed that simulation training is a good learning environment and preparation for acute situations with newborns when the training focuses on strengthening confidence and safety in newborn resuscitation.

#### Spinal reflex

The midwives described a “spinal reflex” in simulations of newborn resuscitation. A midwife mentioned that what they learned in simulation training became a “spinal reflex,” like a combination of experience-based learning from acute situations. One midwife described the following:

*Because you have practised in situations*,* it sticks in the spinal cord when it arises when you have to … even though you are stressed*,* you act according to what you have been trained to do (Informant 3).*

This quote illustrates that the midwives could experience and learn more through simulation and that elements emerged that they were unaware of. The simulation was integrated into their “spinal cord” regarding what they should do in acute situations. The more they trained, the more confident they became in their knowledge and, thereby, performance.

#### Performance anxiety and vulnerability

The midwives spoke about their experience of performance anxiety of being assessed in a simulation situation, which made them feel vulnerable. Therefore, the midwives believed that being as well-equipped and prepared as possible for such situations was essential. A midwife described it as follows:

*It can be scary and make you feel a bit nervous as you enter the simulation*,* but when it’s finished*,* you can almost feel that you fear it less (Informant 5).*

The midwives experienced that it was essential to know what they needed to know and what to do in acute situations, which reduced the stress associated with such situations. A midwife described it as follows:

*It is terribly awkward to do these simulations*,* right*,* and you fear it*,* but it does something with the stress of real situations. THAT stress is reduced. You feel safer*,* which is a big victory when you have completed those trainings too (Informant 6).*

Similarly, this quote is supported by another midwife who described:

*The kind of responsibility you have with a sick newborn baby on the resuscitation table is one of the toughest we have as midwives*,* so in that way*,* I believe we as humans are quite vulnerable (Informant 7).*

The midwives emphasised the importance of confidentiality during simulation training. Focusing on learning and improvement provides emotional safety, which is essential for the experience and learning process. A midwife described the following:

*It is crucial that one feels safe about it*,* confident that confidentiality is maintained*,* and also*,* during simulation*,* that if mistakes are made*,* they should not be talked about. It is allowed; that is the whole point of training. That is where the potential for improvement is*,* to be able to do something about it (Informant 9).*

The midwives reported that they and their colleagues fear simulation training because they do not know the skills well enough and are making a fool of themselves. Another midwife described simulation training as vulnerable:

*It is the same as in reality; you feel vulnerable when you are alone with a non-breathing newborn baby*,* and it does not get any easier when you know there is a group of people watching you (Informant 7).*

This thought-provoking reflection on vulnerability related to handling a sick child, even in a simulated setting, illustrates how the feeling of vulnerability can affect the management of acute situations in newborn resuscitation. They emphasise the importance of a supportive and safe environment to reduce vulnerability and improve professional growth.

### Clear and concise communication- the path to mutual Understanding and improved team interaction

According to the midwives, clear communication was highlighted as central to all collaboration and a fundamental element in simulation training.

#### Clarity

Several midwives experienced clarity as crucial for the team to function effectively and vital for collaboration. Some midwives experienced that the team had improved their ability to be clear and communicate better. In contrast, others reported that only some listened, perceived everything said, or needed to be more explicit. A midwife described it as follows:

*The clarity and who does what is important (Informant 1).*

These descriptions clarify that the midwives mentioned good communication as key to improved teamwork and patient care.

#### Good communication skills

The midwives experienced that communication was time-consuming and a challenging skill to learn. They mentioned that it was essential to implement communication skills in simulation and team training. They talked about how communication skills were what they were least good at and where they could have done better in real newborn resuscitation. A midwife described the following:

*The advantage of getting to practice is that you become aware of your communication skills (Informant 10).*

Good communication skills were discussed as vital for effective collaboration, mutual understanding, and successful newborn resuscitation. Furthermore, they mentioned their insight into how improved communication can be crucial for optimal performance of newborn resuscitation and safe patient care.

#### Person-dependent

Some midwives discussed that good communication depended on whether the team was familiar or unfamiliar and was person-dependent. Others mentioned that communication could be the most challenging aspect in acute situations, but on the other hand, it could also be the easiest. They talked about how it depended on the individual team members. A common goal for everyone in the team was described by a midwife as follows:

*Not everyone becomes equally good*,* but everyone can get a little better (Informant 4).*

Recognising diversity and varied levels of competence, combined with the understanding that everyone has room for improvement, was emphasised by the midwives that individual growth and improvement were attainable for all.

## Discussion

This study highlights the importance of comfort within a familiar team, emphasizing emotional safety, positive learning experiences, and clear communication as key factors for effective collaboration in simulation and team training for newborn resuscitation.

The midwives had a variety of mixed experiences with simulation and team training, but all our informants recommended it as preparation for newborn resuscitation. Simulation was considered a positive preparation for acute situations. However, our findings suggest that learning can be reduced when the situation becomes too safe, and the team may become less alert. Our study shows that focusing on confidentiality and one’s potential for improvement positively affects learning and performance. Emotional safety is vital in simulation and team training to prevent the experience of performance anxiety and vulnerability. Clear communication and mutual understanding are fundamental to all collaboration and essential in simulation and team training. Communication is person-dependent, however, everyone can still improve. Our findings suggest that simulation and team training are good learning arenas that create safety.

### Strengthening team security – a core factor for quality and improved collaboration

Our study indicates that familiarity with the team promoted an excellent and safe learning environment, which was significant in acute situations and during simulation and team training for newborn resuscitation. We understand that simulation training in the same environment provides participants with a sense of safety and control, making it easier to practice various skills, knowledge, and leadership [29]. Similarly, another study suggested that safety and trust are essential in team training across all professions and that getting to know each other is the key to better team training [12].

Therefore, a familiar team and team collaboration are significant for training in newborn resuscitation, which aligns with research indicating that team training improves patient safety [20] Team training should be emphasised in newborn resuscitation [12, 16, 23]. However, it can be more challenging to provide honest feedback to familiar colleagues in the team without risking it being perceived as criticism. Based on findings from a recent study, we found that providing constructive feedback without judgment requires deliberate practice and skill refinement [22]. The midwives in our study experienced that insecurity in role distribution could affect the rest of the team, suggesting that each team member must take responsibility for maintaining their own role and be prepared for acute situations. On the other hand, this preparation must be done in collaboration to synchronise the team.

The midwives emphasised that the team’s composition was necessary for the training, and several midwives experienced it as person-dependent. They described that a team member could be so weak that the rest of the team did not function. Some midwives believed that an unfamiliar team could work well. If the team was unfamiliar with each other, the personality and experience of each team member mattered. We understand this to mean that the team’s behaviour in acute situations can vary based on the team’s composition and provide a picture of group behaviour in a resuscitation [29]. Similarly, another study supports this by demonstrating that simulation-based learning enhances adaptability in team dynamics, allowing healthcare professionals to function effectively even in unfamiliar team settings [10].

The findings suggest that it was essential to experience safety, be coordinated, understand role distribution, know each other’s knowledge and skills, and collaborate well. We interpret this as positive for the training and further collaboration. Team collaboration is an essential prerequisite. Mutual respect and the ability to utilise individual strengths and weaknesses contribute to better team training, thus ensuring good patient safety. Good cohesion creates a positive, stimulating, and constructive learning environment that promotes well-being and is appropriate for the team’s objectives [30]. The concept of cohesion and its importance for the learning environment and the team’s effectiveness is central to simulation and team training in newborn resuscitation [9].

### Preparation and development – the key to continuous growth with bridging to clinical practice

The informants describe the midwives’ responsibility for newborn resuscitation as a burden that makes midwives vulnerable. Contributing factors to negative consequences for newborns include lack of knowledge and skills in basic newborn resuscitation, poor collaboration, and poor communication within the team [16]. Consequently, being well-prepared for such scenarios is crucial, as acute situations are often unpredictable. Our findings align with previous research, concluding that midwives who are vulnerable due to their responsibilities need support and affirmation in their preparations for newborn resuscitation [31].

Therefore, a safe learning environment and a supportive culture are equally crucial for clinical midwives and midwifery students [8, 12]. We interpret this as indicating a need for increased emphasis on psychological safety without fear of being judged negatively and experiencing guilt and shame [32]. The World Health Organisation Report (2018) supports this by emphasising that structured simulation training enhances psychological safety, fostering a more effective learning environment and improving patient care [8]. According to Mesel (2014), the focus should not be on finding scapegoats but rather on promoting collective resilience, where the team takes responsibility for supporting any potential scapegoat, strengthening coping mechanisms, and preventing a scapegoat mentality [32]. We interpret this as the feeling of inadequacy that can lead to a negative experience in simulation training, thus highlighting the importance of emphasising safety, shared responsibility, and the potential for improvement when mistakes occur [8, 21].

Simulation training is an effective learning method that enhances learning and strengthens team collaboration [33, 34]. Moreover, simulation-based education is an effective pedagogical approach that enhances skills and competencies [9, 25]. We interpret this as the simulation training provided the midwives in our study with confidence in acute situations, where they acted based on what was learned and practised, described by the midwives as a “spinal reflex.” We found that simulation and team training helped the midwives identify the necessary knowledge and skills in newborn resuscitation, which contributed to reducing stress in acute situations. A recent study found that in situ simulation, that is, simulation in a clinical setting, supported the students’ learning process, linked theory and practice, and contributed to increased confidence in performing clinical skills [35]. Furthermore, another study concluded that simulation training aims to acquire practical skills and to incorporate and internalise these skills, thereby establishing what our informants called a “spinal reflex” [31]. Moreover, findings from a recent study indicate that frequent simulation training is recommended for effectively developing essential skills in simulation-based education [15].

Clear guidelines and shared responsibility in newborn resuscitation are essential for midwives to avoid emphasising personal blame and to direct focus towards the potential for improvement rather than mistakes [21]. In the context of learning and improvement, this can be understood through the lens of “pedagogy for the unforeseen”, which involves three key components: the degree of familiarity, the level of alertness, and the escalation of the process for identifying the situation [36]. Moreover, this aligns with a study on learning functions highlighting reflective learning, skills training, training in team training, and a systems approach to address errors rather than individual mistakes [22, 23]. However, this is also in line with increased safety and self-confidence, reduced stress levels, and improved team training, which are essential in simulation and team training [8, 9, 17, 23]. A recommendation for simulation training and tailored training to be conducted as part of a lifelong learning process from the beginning of education and throughout the entire professional career as preparation for newborn resuscitation [12] is a similar finding to our study. Furthermore, regular brief simulation sessions can improve and maintain skills in ventilating newborns in an interdisciplinary setting and contribute to quality improvement in newborn resuscitation [15, 19, 37]. Aligned with the studies mentioned above, midwives in our study suggested low-dose, high-frequency simulation training with frequent and brief sessions to ensure that there is only a little time between each training session [10].

The midwives in our study stressed the importance of structuring simulation training to reflect the realistically available competence during different shifts, as acute situations in some maternity wards often involve fewer staff and limited expertise. We interpret this as simulation being a valuable and good pedagogical tool due to the presence in the situation and dilemmas made visible with the opportunity to explore the consequences of incorrect assessments. Furthermore, this matter reduces the distance between theory and practice [25, 29], and thereby contributes to bridging a gap. This insight underpinned the necessity to consider improving and further developing simulation training to enhance learning and understanding by building bridges between theoretical knowledge and practical execution. The world Health Organization Report (2018) similarly highlights the importance of structured simulation training in nursing and midwifery education, reinforcing its role in bridging theoretical knowledge with practical execution [8].

### Clear and concise communication – the path to mutual Understanding and improved team interaction

Our findings show that clear communication is vital for effective team functioning and collaboration. The midwives emphasized the importance of clear and good communication skills within the team but noted that these skills often depend on the individual. This suggests that team dynamics vary, with some members excelling in collaboration, actively participating, and assuming leadership roles, while others may take a less prominent approach [30]. Our study revealed that communication was a challenging and time-consuming skill to master, but it was also a crucial component in simulation and team training. The midwives experienced that communication was their weakest area and where they most often failed in actual events. Simulation training improved clarity and communication in both simulated training and acute newborn resuscitation. We found that communication seemed dependent on the individual, regardless of whether the team was familiar or unfamiliar. We interpret this as communication skills that can be improved by focusing on active listening, confirmation of nonverbal and verbal language, questioning, information dissemination, and reflection [30]. Another study further emphasises that simulation training strengthens team cohesion, improving communication and collaboration in newborn resuscitation scenarios [9].

Several studies support our findings, highlighting the importance of clear communication within the team and what can improve communication skills in newborn resuscitation [12, 16]. Another study pointed out that factors such as hierarchy and leadership are experienced positively and make team training easier [24].

Moreover, a study shows that mutual understanding, distribution of tasks and responsibilities with a focus on language and good communication skills are essential [38]. Our findings suggest that lack of communication can be person-dependent. This means the team must be aware of and utilise each other’s strengths and weaknesses to improve team training. Therefore, trust, openness, and equality are essential factors in the team’s collaboration. The team process must also allow for differences, recognition, support, and room to give praise to each team member [30]. A common understanding and clear communication, reinforced by good communication skills, are crucial for both team training in simulated scenarios and the performance of real newborn resuscitation [8, 9].

### Strengths and limitations

A strength of our study is the diverse range of midwives’ experiences, spanning different ages and levels of clinical expertise, ensuring reliability and credibility [27]. We included all qualified participants in the study for credibility [27]. We used a qualitative method with an explorative design and thematic cross-sectional analysis, which was well-suited to exploring midwives’ experiences in Norwegian maternity wards. Both authors and the supervisor independently and collectively engaged in the analysis to validate our findings, supported by direct participant quotes [27]. Another strength, which can also be a weakness in our study, is that we both have extensive experience as midwives in the clinic, which means that we both have a pre-understanding of the topic we are researching [26]. Including maternity wards with and without neonatal intensive care units strengthened the study. However, the voluntary participation may have introduced a bias towards those with a particular interest in the topic. Nevertheless, our findings contribute valuable knowledge on simulation and team training, and the detailed methodology allows for replication of the study.

### Implications for practice

The findings contribute increased insight and provide an enhanced understanding of midwives` simulation experience in team training in newborn resuscitation, pointing to simulation training and team training as good learning arenas in supportive and safe environments. This implies that, in practice, we recommend conducting low-dose, high-frequency simulations in supportive and safe environments with clear communication. Such simulations can foster development, enhance trust and collaboration, improve the working environment by reducing stress and increasing well-being, minimise misunderstandings, facilitate effective decision-making, support continuous improvement, and promote a robust feedback culture. Furthermore, extensive training is recommended to ensure that both technical and non-technical skills are maintained and further developed. The structure of simulations should aim to be implemented consistently across Norway.

### Further research

Further research on the experience of simulation and team training could be beneficial for practising and preparing newborn resuscitation in maternity wards in Norway. It may also be relevant to pursue further research on the working environment and its impact.

## Conclusion

Our study highlights the critical importance of fostering psychological safety within familiar teams, emphasising how emotional security contributes to effective learning and professional growth. The findings demonstrate that recognizing emotional safety leads to more positive learning experiences, fostering competence and confidence in clinical practice. Furthermore, clear communication is identified as essential for collaboration, ensuring beneficial experience in simulation and team training related to newborn resuscitation. By strengthening team communication and psychological security, simulation training not only enhances theoretical understanding but also directly impacts clinical decision-making, patient outcomes, and team dynamics in acute newborn resuscitation scenarios.

## Data Availability

The dataset used and analysed during this study is available from the corresponding author upon reasonable request.

## Declarations

### Declaration of generative Al and Al-assisted technologies in the writing process

Statement: During the preparation of this work, the authors used Chat UiT to revise the text after it was completed; this was done to improve the readability and language of the manuscript. After using this tool, the authors reviewed and edited the content as needed and took full responsibility for the content for publication.
